# Effective Ki-67 Reduction by Pre-Surgery Short-Term Administration of Letrozole in (Hormone-Positive) Breast Cancer

**DOI:** 10.30699/IJP.2023.2002772.3118

**Published:** 2023-12-29

**Authors:** Bita Eslami, Sadaf Alipour, Farzaneh Golfam, Behnaz Jahabnin, Ramesh Omranipour

**Affiliations:** 1 *Breast Diseases Research Center, Cancer Institute, Tehran University of Medical Sciences, Tehran, Iran*; 2 *Department of Surgery, Arash Women's Hospital, Tehran University of Medical Sciences, Tehran, Iran*; 3 *Mostafa Khomeini Hospital, Shahed University, Tehran, Iran*; 4 *Department of Pathology, Cancer Institute, Imam Khomeini Hospital Complex, Tehran University of Medical Sciences, Tehran, Iran*; 5 *Department of Surgical Oncology, Cancer Institute, Tehran University of Medical Sciences, Tehran, Iran*

**Keywords:** Breast cancer, Ki-67 Antigen, Letrozole, Postmenopause

## Abstract

**Background & Objective:**

Antigen Ki-67 (histone-based nuclear protein) is a static marker of tumor cell proliferation and growth and is commonly measured to indicate the effect of treatment in breast cancer patients. This single-arm trial study aimed to evaluate the effect of short-term endocrine therapy (letrozole) on Ki-67 levels in menopausal women with early hormone-positive breast cancer who were referred to two university hospitals.

**Methods:**

Patients with a pre-treatment Ki67 of 5% or less were excluded from the study. Participants (n=25) received oral letrozole (2.5 mg daily) seven days before surgery. Ki-67% on both biopsies and the surgical specimens were measured and compared.

**Results and Conclusion:**

The mean age of patients was 62±9.4 (48-83 years). Our result indicated that pre-surgery consumption of letrozole for hormone-positive breast cancer can significantly decrease the of Ki-67 labeling index (23.24±9.74 vs. 16.92±9.55, *P*=0.001 by paired t-test), with no drug-related adverse events.

## Introduction

Breast cancer is the most common type of cancer and the second leading cause of cancer mortality (1). Early breast cancer has a highly variable prognosis, and the results of neoadjuvant endocrine therapy in this type of cancer are unpredictable (2). Tumor size, tissue grade, vascular invasion, and lymph node involvement status are known factors in determining the prognosis of this type of disease (3). Estrogen receptor and Her-2 neu are now considered predictors of response to treatment and prognosis of patients (4).

Antigen Ki-67 (histone-based nuclear protein) is a static marker of tumor cell proliferation and growth. It is commonly measured to predict the effect of initial treatment and the expected prognosis in long-term follow-up patients (5). Ki-67 can be used as a dynamic marker to indicate the effect of treatment, and the association of Ki67% and response to the treatment was evaluated in several studies. Some studies reported the higher value of ki67% in early or locally advanced breast cancer was associated with better response to chemotherapy (6-9). In contrast, some evidence didn't find any relationship between Ki67 labeling index (LI) and response to neoadjuvant endocrine treatment (9-11). Due to the fact that Ki-67 is easy to measure, has a low cost, and can be easily repeated, evaluation of its fluctuations during treatment phases can be helpful and cost-effective (4, 12).

Today, the combination of systemic therapy and surgery has significantly increased the survival of breast cancer patients. Endocrine therapy is one of the main systemic treatments for hormone-positive tumors, especially in the early stages of the disease; (13) but despite the known advantages of this type of treatment, more than 20% of the patients discontinue their treatment (14). Some patients also experience anxiety in the interval between diagnosis and surgery due to the inevitable postponement of their treatment secondary to the busy schedules in public hospitals; these patients often request some type of treatment during this gap. Therefore, some studies have investigated whether different medical treatments could be used during this lag; they have assessed Ki-67 LI to estimate the effect of their treatment (12, 15, 16).

The present study aimed to find out whether short-term endocrine treatment with letrozole can decrease the Ki-67 LI in postmenopausal early-stage hormone-positive breast cancer.

## Material and Methods

This single-arm trial was performed on breast cancer patients who were referred to the Cancer Institute of Imam Khomeini Hospital Complex and to Arash Women's Hospital, Tehran University of Medical Sciences (TUMS), Tehran, Iran, between April 2019 and March 2020. Menopausal women with early hormone-positive breast cancer were recruited for this study. The breast cancer and the tumor subtype were diagnosed by histological assessment and immunohistochemistry (IHC) using a core needle biopsy (CNB) specimen taken with a 14-gauge needle. Ki-67 LI on both biopsies and the surgical specimens were measured. Patients with a Ki-67% of 5% or less were excluded from the study. The principal investigators explained the study protocol to the patients. Participants then received oral letrozole (2.5 mg daily) for seven days before surgery. After that period, surgery was carried out, and the specimen was sent for histological examination and IHC. The results were recorded and included tumor size, tumor grade, Her2 status, and Ki-67 LI . Patients' data, such as age, weight, height, side of cancer, grade, HER2 status, and type of surgery, were retrieved from medical profiles. 

Written informed consent was obtained from all patients prior to entry into the study, and the ethics committee of TUMS approved this study (IR.TUMS.VCR.REC.1398.966). This was a clinical trial study that received an IRCT code (IRCT20101104005101N2). Data were analyzed using SPSS 22 software (SPSS Inc., Chicago, IL., USA).

## Results and Discussion

In the final analysis, 25 out of 40 (63%) patients had both Ki-67% of needle biopsy and surgery results. The mean age of patients was 62±9.4 (48-83 years). All patients were diagnosed with invasive ductal carcinoma. Regarding the type of surgery on the breast, 68% underwent breast conservative surgery, and a mastectomy was performed on the rest of them. Table 1 shows the total characteristics of the 25 cases. Comparison of Ki67 LI before and after using letrozole shows a significant decrease (23.24±9.74 vs. 16.92±9.55, *P*=0.001 by paired t-test). Treatment with letrozole caused no drug-related adverse events. 

**Table 1:** Total characteristics and Ki-67 LI changes in 25 patients

Continuous Variables						
		**Min**	**Max**		**Mean**	**SD**
Age (yrs)		48	83		62	9.14
BMI (Kg/m^2^)		25.39	31.25		27.65	1.94
Ki-67 of the biopsy specimen (%)		7	40		23.24	9.74
Ki-67of the surgical specimen (%)		3	30		16.92	9.55
Categorical Variables						
	**Number**			**Percentage**		
Breast Side Right Left	15 10			60 40		
Tumor Grade 1 2 3 Unknown	5 16 1 3			20 64 4 12		
Axillary lymph node involvement Yes No Unknown	7 13 5			28 52 20		
Ki-67 Changes Decreased level Increased level Without changes	15 5 5			60 20 20		

Min= Minimum; Max= Maximum; SD= Standard deviation.

Our study result indicates that a 7-day period of consumption of letrozole before surgery for hormone-positive breast cancer can significantly decrease the Ki-67 LI in menopausal women. 

In a study by Dowsett *et al.*, Ki-67 LI was measured before and after 2 and 12 weeks of administration of anastrozole and tamoxifen, alone or in combination. They found that suppression of Ki-67 activity was significantly greater with anastrozole than with tamoxifen (12). A further study found that higher Ki-67 LI after 2 weeks of endocrine therapy before surgery with anastrozole or tamoxifen or the combination of anastrozole plus tamoxifen was statistically significantly associated with lower recurrence-free survival. In contrast, higher Ki67 expression at baseline was not (17).

Iwamoto *et al.* showed that two weeks of endocrine therapy (with tamoxifen (20 mg) in premenopausal patients and letrozole (2.5 mg) in menopausal cases) prior to the breast cancer surgery significantly lowered the Ki-67 LI of the tumor (15). They classified post-Ki67 LI as many low-risk cases as genomic markers and suggested that post-Ki67 LI can be used as a reliable substitute for genomic testing. While genomic testing is likely to improve survival and quality-adjusted life expectancy in patients with high recurrence risks, the cost of genomic testing limits its use, specifically in low-income countries (18, 19). 

Women with untreated stage one or two, estrogen receptor-positive, and invasive breast cancer received 20 mg of tamoxifen for 7 days up to the day of surgery in the study of Cohen et al. The result showed short-term tamoxifen use had a similar relative decrease in Ki-67 LI as that reported for longer courses, and this protocol was acceptable for participating women (16).

In 1994, DeFriend *et al.* conducted a study on the administration of a new antiestrogen drug, ICI 182780, to patients with primary breast cancer. In this clinical trial, 56 patients were selected, of which 19 were controls, and 37 were in the treatment group. In the treatment group, 7 mg (21 patients) and 18 mg (16 patients) of the compound were injected intra-muscularly 7 days before breast surgery. The treatment significantly reduced hormone receptors and Ki-67 gene expression in patients with hormone-positive tumors. This treatment was well tolerated, and the antiestrogenic effects of the medicine occurred without agonist activity (20). Evidence shows that short-term administration of hormone therapy has advantages and is an accessible and inexpensive method with very few side effects. 

The most important advantage of this method was the use of hormone therapy, which is cheap and available for patients, and its results can be easily evaluated. However, the small sample size of this study is an obvious limitation that must be investigated in studies with a larger sample size.

## Conclusion

Overall, our study shows that short-term endocrine therapy for hormone receptor-positive early breast cancer in menopausal patients can effectively decrease Ki-67 LI, a marker of proliferation of the tumor. Considering the findings of the current study are confirmed on a larger scale, patients can benefit from possible advantages of this method. Using letrozole for one week between the last visit and surgery at least may reduce the proliferation rate of the tumor. , In addition, treatment during the waiting period until surgery reduces the anxiety and stress of the patients.

## Funding

None.

## Conflict of Interest

The authors declare no conflict of interest.
